# Bioactivity, hemocompatibility, and inflammatory response of calcium incorporated sulfonated polyether ether ketone on mouse-derived bone marrow cells

**DOI:** 10.1042/BSR20232162

**Published:** 2024-06-26

**Authors:** Shanmuga Sundar Saravanabhavan, Prabhu Narayanaswamy Venkatesan, Narendranath Jonna, Kamalakannan Vasantha Palaniappan, Zsolt Sarang, Balasubramanian Natesan, Consolato M. Sergi

**Affiliations:** 1 Department of Laboratory Medicine and Pathology, University of Alberta, Edmonton, AB, Canada; 2 Department of Biotechnology, Aarupadai Veedu Institute of Technology, VMRF (DU), Paiyanoor, Tamil Nadu, India; 3 Department of Chemistry, Easwari Engineering College, Ramapuram, Chennai, India; 4 Centre for Energy Storage Technology, Anna University, Chennai, India; 5 Department of Biochemistry and Molecular Biology, Faculty of Medicine, University of Debrecen, 4032 Debrecen, Hungary; 6 Division of Anatomic Pathology, Children's Hospital of Eastern Ontario, University of Ottawa, ON, Canada

**Keywords:** Bioactivity, bone, cytokines, Haemocompatibility, inflammation, SPEEK

## Abstract

Natural and synthetic polymeric materials, particularly soft and hard tissue replacements, are paramount in medicine. We prepared calcium-incorporated sulfonated polyether-ether ketone (SPEEK) polymer membranes for bone applications. The bioactivity was higher after 21 days of immersion in simulated body fluid (SBF) due to calcium concentration in the membrane. We present a new biomaterial healing system composed of calcium and sulfonated polyether ether ketone (Ca-SPEEK) that can function as a successful biomaterial without causing inflammation when tested on bone marrow cells. The Ca-SPEEK exhibited 13 ± 0.5% clot with low fibrin mesh formation compared to 21 ± 0.5% in SPEEK. In addition, the Ca-SPEEK showed higher protein adsorption than SPEEK membranes. As an inflammatory response, IL-1 and TNF-α in the case of Ca-SPEEK were lower than those for SPEEK. We found an early regulation of IL-10 in the case of Ca-SPEEK at 6 h, which may be attributed to the down-regulation of the inflammatory markers IL-1 and TNF-α. These results evidence the innovative bioactivity of Ca-SPEEK with low inflammatory response, opening venues for bone applications.

## Introduction

Natural and synthetic polymeric materials have been used for soft and hard tissue replacements. These include chitosan, polyacrylate, polysiloxanes, polysulfone, polyether ether ketone (PEEK), ultrahigh molecular weight polyethylene, polyurethane, polyamides, and polytetrafluoroethylene. PEEK is widely used for load-bearing orthopedic applications, as it is biocompatible and possesses good mechanical properties [1–4]. Many reports suggest using hydroxyapatite as a filler to enhance its bioactivity. However, the primary cause of concern while using hydroxyapatite is its brittle nature and difficulty in processing, making it unsuitable for certain biomedical applications [5–8]. It is also noteworthy that PEEK cannot transfer bone morphogenetic proteins when bones require them and is insoluble in most solvents. These characteristics of PEEK are of major concern since they pose problems if the bone cement loosens up, causing failure of the implant [9]. This insolubility issue has driven the focus of researchers toward sulfonated PEEK (SPEEK), which is soluble in several common solvents, thus making it possible to fabricate SPEEK-based composites in different forms such as fibers, films, and spheres. Many researchers have reported using SPEEK-based biomaterials for drug delivery, vascular grafts, and soft tissue prostheses [10,11].

Several reports have provided evidence for the bioactivity of SPEEK in combination with hydroxyapatite. In addition, some studies have shown promising results in using surface-modified polymers for good bond formation between the sulfonyl groups and hydroxyapatite [12,13]. These composites show enhanced bioactivity, which could be instrumental in developing a bond between the prosthesis and the host bone. This aspect will address the concerns regarding prosthesis failure occurring due to loss of bone cement (through wear) and other factors such as wear and tear, fatigue, fracture of the material, and improper positioning of the implant [14]. Another major cause of concern is the biocompatibility of the prosthetic material. Among the polymers evaluated for implant applications, PEEK, PMMA, UHMWPE, and PEG were less reactive in the body’s environment. Although no synthetic material can be completely biocompatible, it is possible to reduce the severity of the host response through suitable surface modifications and the use of polymer blend [14,15].

Polymer composites for medical applications with blood-contacting surfaces need to be evaluated for hemocompatibility and protein adsorption. In vitro biocompatibility testing is done routinely using various techniques, mainly designed based on the end-use of the polymers. Reports suggest that most hydrophobic polymers adsorb more serum proteins than hydrophilic polymer [16]. Hemocompatibility studies aid in the prediction of host-tissue reactions to foreign implant material. An essential factor is *in vitro* bioactivity. It evaluates the osteoinductive ability of the polymer. It is based on the amount of apatite layer formed on the surface of the membrane after immersion in simulated body fluid [17]. In the present study, we fabricated Ca-SPEEK from SPEEK and analyzed its bioactivity over 21 days. To evaluate whether the material is suitable as a blood-contacting biomaterial, we investigated the hemocompatibility and protein adsorption of SPEEK and Ca-SPEEK. Bone marrow-derived macrophage cells were used to study the inflammatory response of the polymer membranes.

## Materials and methods

Medical-grade PEEK powder was procured from Victrex, England, U.K., while sulfuric acid and calcium hydroxide were sourced from Merck, India. Other chemicals and solvents, such as N-Methyl Pyrrolidone (NMP), PBS, trypsin, and those required for preparing SBF, were procured from SRL, India. The chemicals procured were used as received from the manufacturer with no modifications.

The experiments involving mice for bone marrow cell isolation were performed according to the instructions and guidelines issued by the University of Debrecen (DEMAB) Ethical Committee for animal care. Mice were bred under specific pathogen-free conditions in the central animal facility of the University of Debrecen. Mice were anesthetized with 2.5% isoflurane using a SomnoSuite device. The euthanasia was performed by the same Somnosuite device. Only thing that we increased was the isoflurane concentration to 5%. The mice just died of respiratory failure. The experiments were performed as per the animal experiment certificate number and Ethics Approval # 7/2016/DEMÁB at University of Debrecen, Debrecen, Hungary. Ethics approval was also granted and hemocompatibility human experiments were performed in accordance with the World Medical Association Declaration of Helsinki, and all subjects provided written informed consent. A copy of this consent can be obtained from the senior author at the University of Ottawa, Ottawa, Ontario, Canada. The blood samples used in the study were drawn from healthy individuals at random with their consent. The materials were tested for clot studies on blood samples drawn from healthy volunteers with their consent. There were no in vivo studies involving human or animal models reported in the present study.

### Preparation of SPEEK membranes

As described in our previous work, PEEK was sulfonated using sulfuric acid [10,11]. Briefly, 3 g of dried PEEK were dissolved in 45 ml of sulfuric acid and stirred continuously for 7 hours under a nitrogen atmosphere. The reaction was then terminated using ice-cold water, and the degree of sulfonation obtained was 50%, as interpreted from the ^1^H NMR results reported in our previous study [18]. To remove the excess acid present from the precipitated SPEEK, it was washed several times with deionized water until the pH reached 7.0 and then dried. After that, the SPEEK, dissolved in NMP (2 g in 250 ml), was cast on a clean, dry petri dish, and the solvent was allowed to evaporate at room temperature, resulting in the formation of a SPEEK membrane with a thickness of 0.8 mm. The membrane was carefully separated from the Petri dish for further use. The sulfonation of PEEK using sulfuric acid is shown schematically in Fig. S1.

### Preparation of calcium doped SPEEK membranes

The membrane was cut into 1 cm^2^ piece and immersed in a saturated calcium hydroxide (CaOH)_2_ solution at room temperature for 24 h. Ca(OH)_2_ saturated solution was made by dissolving 0. 824 g of it in 1 L of water while constantly stirring. The exothermic reaction was allowed to proceed for 3 h, after which the solution was filtered, and the filtrate was used for the study. The membranes were retrieved and left to dry overnight, after which they were characterized using Fourier-transform infrared spectroscopy (FTIR), which is a technique used to obtain an infrared spectrum of absorption or emission of a solid, liquid, or gas. FTIR was used to confirm the formation of the calcium-sulfonic (SO_3_H) group bond. FTIR analysis was conducted on the dried membrane using a Perkin Elmer Spectrum RXI IR spectrophotometer at 25 ± 2°C. Initially, the sample to be tested was dried at 100°C for an hour. The spectra were recorded to identify the presence of sulfonic groups in SPEECH. The schematic representation of calcium-incorporated SPEEK is shown in the Supplementary Materials (S1). Next, the prepared samples were analyzed using an optical polarising microscope (OPM) (Labomed, LX-400) for surface changes on the SPEEK membrane (S2 in Supplementary Materials). Finally, the membranes were used for further studies.

### Determination of swelling

The water absorption property of the membranes was evaluated by soaking the pre-weighed dried membranes (of size 1 cm^2^) in deionized water for 24 h. The membranes were retrieved, and the excess water was removed from the membrane surface using blotting paper. The wet weight of the samples was weighed accurately. The water uptake of the membrane determines its suitability and application. The incorporation of hydrophilic groups or functionalization and the addition of fillers can modify this property. The percentage of swelling was calculated using the following formula:

$$\%\;Water\;absorption=\frac{Weight\;of\;wet\;polymer}{Weight\;of\;dry\;polymer}*100$$

### Bioactivity

For the bioactivity study, simulated body fluid (SBF) was prepared in the lab as per our previous studies [10,11] and the procedure described by Ayako Oyane et al. [19]. To evaluate the bioactivity, SPEEK, and Ca-SPEEK membranes were immersed in SBF for varying periods (0, 7, 14, and 21 days) to analyze the apatite growth formation. We used a SEC high-resolution scanning electron microscope (Model No.: SNE-4500M Plus(B)) and a Bruker XFlash® 600 Mini silicon drift detector to confirm apatite growth or bioactivity on the membrane surface.

### Hemocompatibility studies

The hemocompatibility studies were carried out by immersing membranes of size 0.5 mm^2^into the blood samples. The procedure was described in one of our previous studies. In this study, the protocol described in our early study was followed [2], with the HITACHI S-3400N Scanning Electron Microscope (SEM) used to examine the immersed membrane for detailed fibrin formation. In addition, the weight of the blood clot formed against SPEEK and Ca-SPEEK was measured.

### Protein adsorption

For the protein adsorption test, 1.2 g of trypsin were dissolved in 100 ml of PBS with a pH of 7.6. The prepared membrane samples of SPEEK and Ca-SPEEK were immersed in the trypsin solution for 24 h. Protein adsorption was determined using the PerkinElmer Spectrum RXI IR spectrophotometer, SEC High-Resolution Scanning Electron Microscope (Model No. SNE-4500M Plus (B)), and Bruker XFlash® 600 Mini silicon drift detector [20].

### Inflammatory response

When introduced *in vivo*, the polymer membrane could elicit an inflammatory reaction; hence, we used mice’s bone marrow-derived (BMDMSs) macrophage cells to study this aspect. The methodology used for the study was adapted from our previous study [21]. With an approximate weight of 25 g, mice were sacrificed and purified using ethanol femurs to avoid disinfection. After purification, the bone marrow was harvested, and the cells were allowed to differentiate for five days in T75 flasks for further studies. The membranes (SPEEK and Ca-SPEEK) were placed in the wells of a 6-well plate and studied for 6 and 24 h to see how they affected the regulation of inflammatory markers like IL-1, IL-10, and TNF-α. In addition, a microscopic evaluation was used to identify the morphological changes associated with incorporating these membranes into the cell lines. Bone marrow-derived macrophage cells (BMDMS) were seeded on to two 6-well plates at 2 × 10^5^ cells/well density in Dulbecco’s Modified Eagle Medium (DMEM) and 10% FBS and incubated for 24 h. IL-1, IL-10, and TNF-α were measured to assess the inflammatory response using qPCR in BMDMS cells after 6 and 24 h of membrane treatment [21]. Catalog numbers of the TaqMan assays (ThermoFisher Scientific) used were the following: Tnf Mm00443258_m1, IL1B Mm00434228_m1, IL10Mm01288386_m1, and Actb Mm02619580_g1. The experiments throughout the study were carried out in triplicate, and the results were plotted using Origin Pro software version 8 (OriginLab, Northampton, MA, U.S.A.).

### Statistics

All data presented represent the result of at least three independent experiments, and all data are presented as mean ± SD. Statistical analysis was performed using a two-tailed, unpaired Student’s *t*-test. The equal variance of the sample groups was tested by F-test. * denotes *P*<0.05.

## Results

### Bioactivity

**Figure 1:**
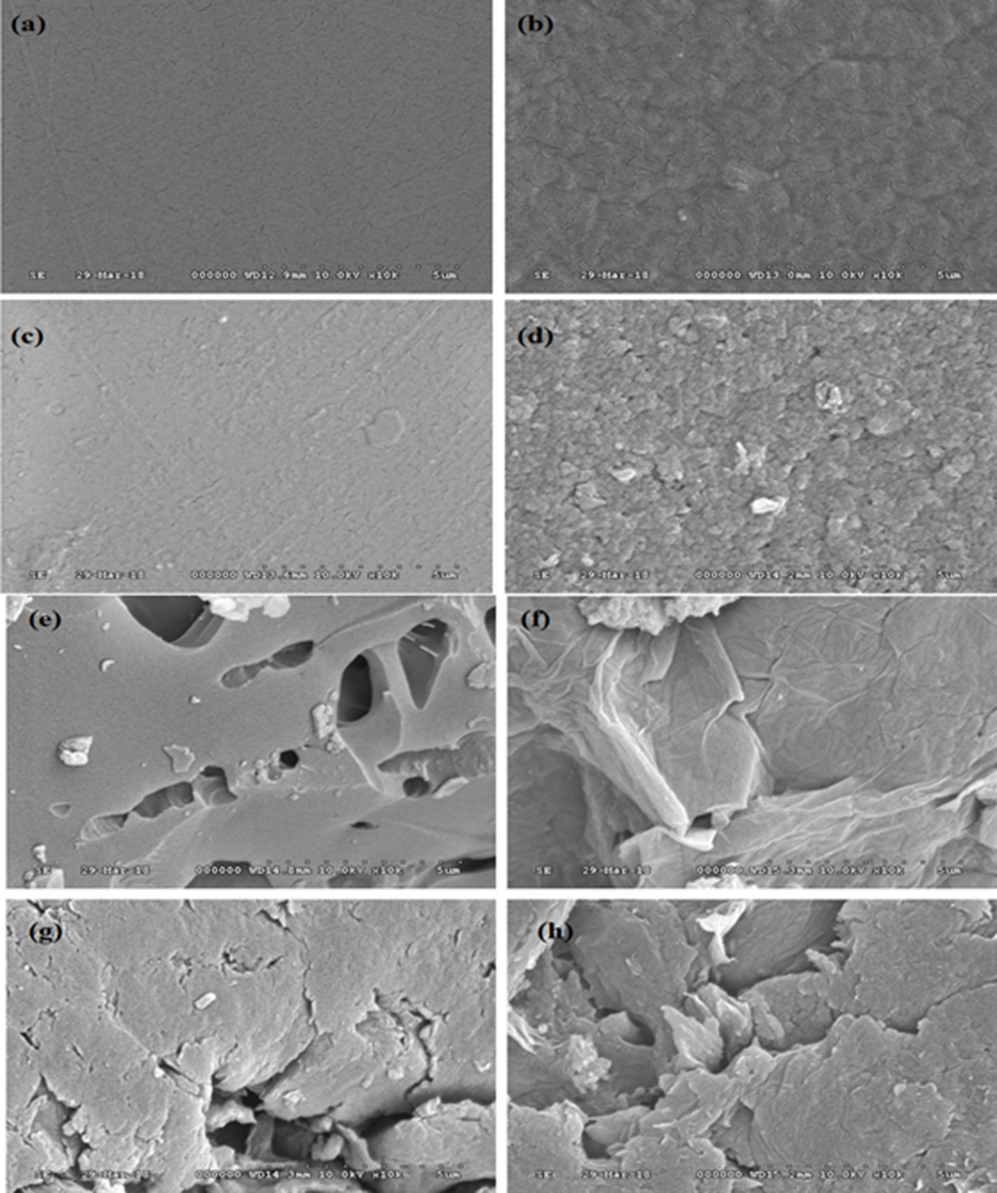
Scanning Electron Microscopy (SEM) images of the mebranes before and after immersion in simulated body fluid (SBF) solution SEM images correspond to the membranes before and after immersion in SBF solution, where (**A**) SPEEK membrane before immersion in SBF solution, (**B**) SPEEK membrane after 7th day of immersion in SBF showing the start of apatite formation, (**C**) SPEEK membrane after 14th day of immersion in SBF shows the extension of apatite growth, (**D**) SPEEK after 21st day of immersion in SBF indicating the apatite growth, (**E**) Ca-SPEEK (0 days in SBF), (**F**) Ca-SPEEK after 7th day of immersion in SBF clearly indicates the high amount of apatite growth compared with SPEEK membrane due to the addition of Ca, (**G**) Ca-SPEEK after 14 days of immersion in SBF and (**H**) Ca-SPEEK after 21 days of immersion in SBF showed apatite deposition on the membrane; this rate of transformation was ascertained to be due to the presence of Ca, which was absent in the SPEEK membrane.

SEM was used to examine apatite formation on prepared SPEEK and Ca-SPEEK membrane samples after immersion in SBF solution. Figure 1 shows the SEM images of SPEEK retrieved from SBF after 0, 7, 14, and 21 days of immersion. The SEM images in Figure 1A–D show that the SPEEK surface before and after immersion in SBF exhibited no apatite formation in all four cases. The roughness of the membrane was observed to be increasing slightly, which might be due to the attraction of the ions in the SBF solution. The Ca-incorporated SPEEK membrane also displayed similar findings during the 0th day, as shown in Figure 1E. However, after immersion of the membranes in SBF for 7 days, SPEEK exhibited less dense or no apatite formation when compared with that of Ca-SPEEK-based membranes (Figure 1F). It had dense apatite formation on its surface. It was observed that the growth of apatite increased over time on both Ca-SPEEK (Figure 1G,H) and SPEEK membranes. The water uptake of these membranes was given in Supplementary Table S1.

**Figure 2:**
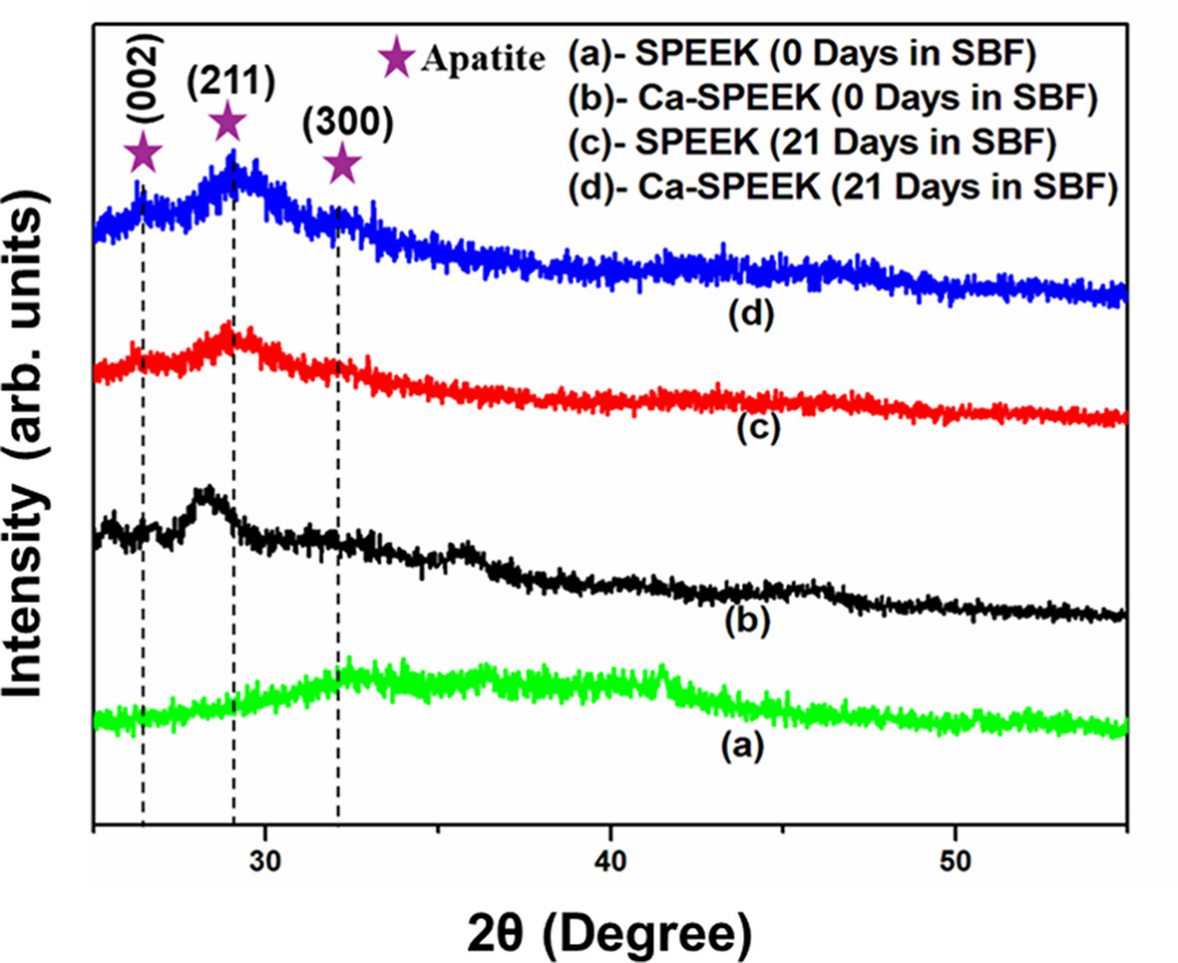
X-ray diffraction (XRD) spectra of sulfonated poly-ether-ether ketone (SPEEK) and Ca-SPEEK at different times XRD spectra of (**A**) SPEEK, (**B**) Ca-SPEEK immersed in SBF during 0 days, (**C**) SPEEK and (**D**) Ca-SPEEK after immersion in SBF after 21 days (see text).

Additionally, X-ray diffraction (XRD) patterns of SPEEK and Ca-SPEEK after the 0 and 21 days of immersion in SBF were studied (Figure 2), wherein the 0th day was considered the control sample. From Figure 2, intensities with several diffraction maxima corresponding to the apatite-like phase (ASTM JCPDS 9-432) were observed. It was clearly seen that the sulfonation and surface modification with Ca had increased the nucleation sites, which translated into enhanced bioactivity (ASTM JCPDS 21-816). Figure 2 shows the XRD spectra of SPEEK, Ca-SPEEK, and their diffraction patterns after immersion in SBF for 0 and 21 days, wherein the 0th day was considered the control sample. Figure 2D shows the characteristic peaks of amorphous SPEEK material. In addition, Figure 2B–D shows the crystalline diffraction peaks of apatite around 26°, 29.5°, and 32°, corresponding to the (002), (211), and (300) planes, respectively. From Figure 2, it was observed that after immersion in SBF for 21 days, both SPEEK and Ca- SPEEK exhibited a dramatic increase in their diffraction intensities with a broadening of peaks, which may be attributed to the formation of more apatite over the SPEEK, which was also evident from SEM analysis. Thus, it is clearly seen that the sulfonation and surface modification with Ca^2+^ have facilitated the nucleation sites, as a result of which the bioactivity has been enhanced.

**Figure 3:**
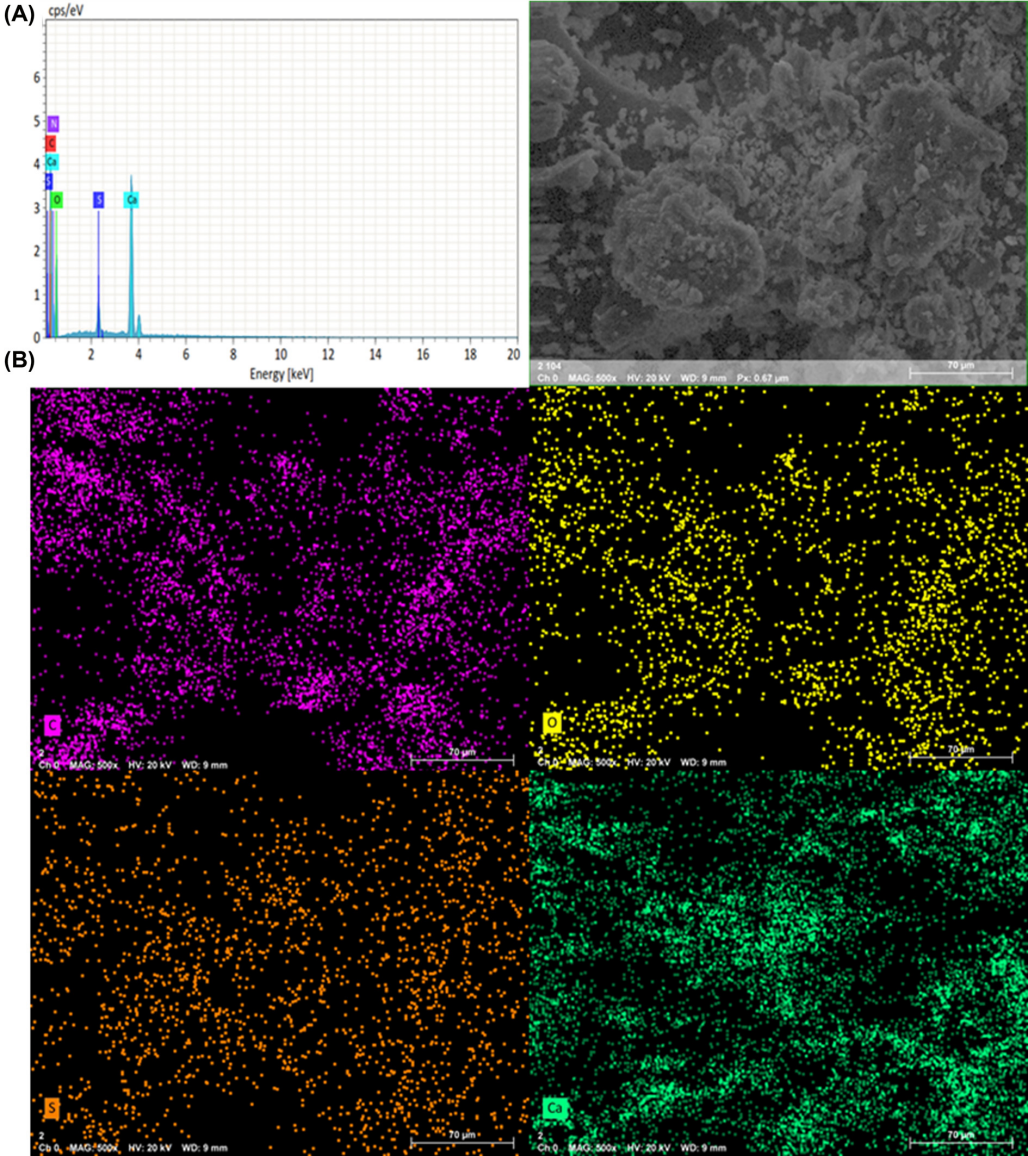
Energy-dispersive X-ray spectroscopy (EDS) of SPEEK immersed in SBF EDS reveals (**A**) spectra and (**B**) mapping of SPEEK immersed in SBF after 21 days of immersion in SBF (see text).

**Figure 4:**
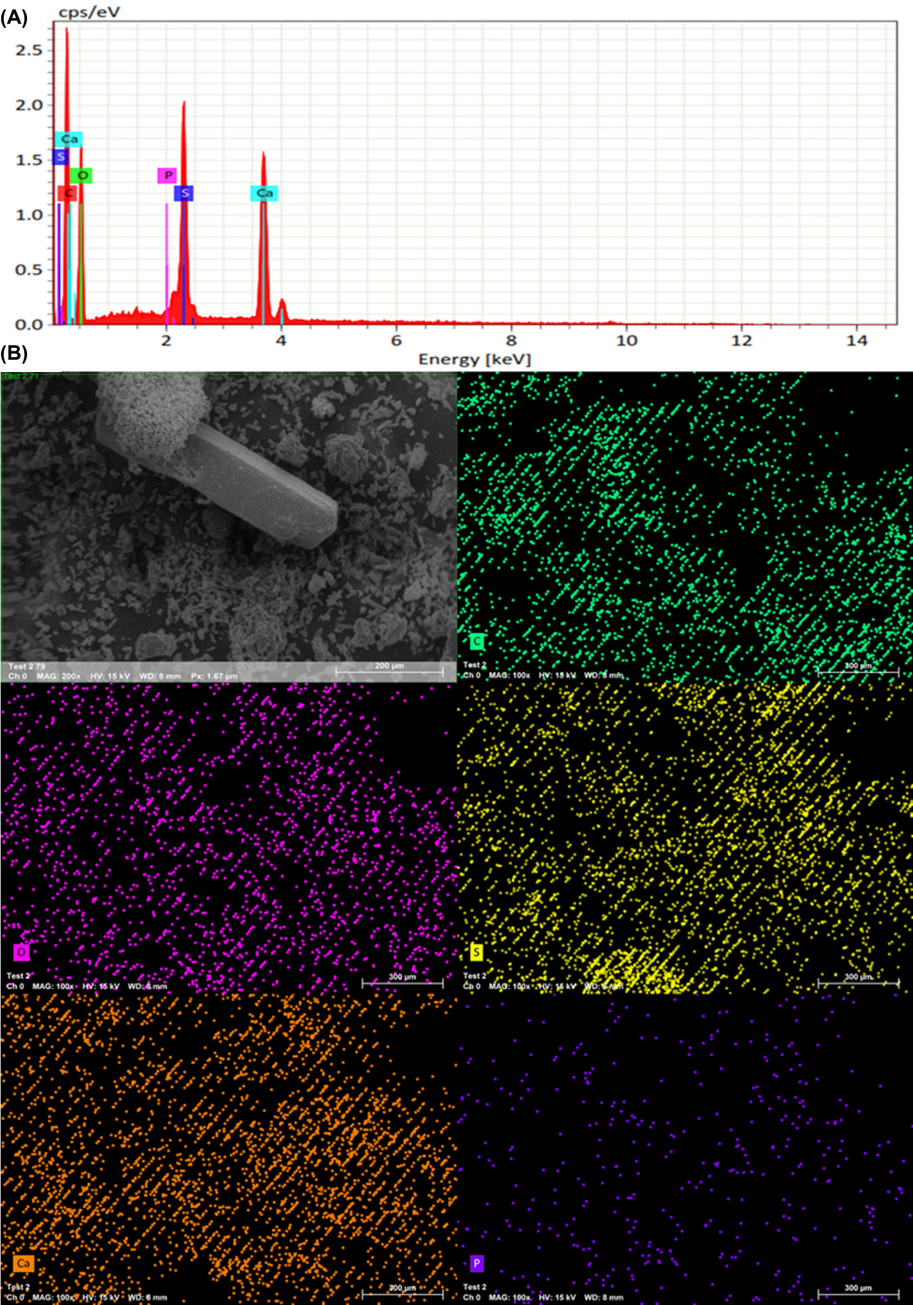
Energy-dispersive X-ray spectroscopy (EDS) of Ca-SPEEK immersed in SBF EDS reveals (**A**) Spectra and (**B**) Mapping of Ca-SPEEK immersed in SBF after 21st day of immersion in SBF.

It was evident that the involvement of the SO_3_H group when combined with Ca improved the efficiency of apatite formation. The intensities were comparatively higher in the case of Ca-SPEEK compared with the typical SPEEK models on the 21st day. This was further supported by the SEM results [12]. The results of the energy-dispersive X-ray spectroscopy (EDS) analysis in Figures 3 and 4 show that the presence of the PO_4_ group was observed with samples immersed in Ca-incorporation, whereas the same was not the case with the SPEEK alone. The EDS spectra of the as-prepared SPEEK and Ca-SPEEK membranes do not indicate the formation of apatite, as shown in Supplementary Figures S3 and S4.

In order to evaluate the mechanical property of the material, tensile strength was evaluated. The results show that the incorporation of Ca-SPEEK has increased the tensile property of the membrane, which may be attributed to the incorporation of Ca^2+^ into the SPEEK. Moreover, the addition of Ca2+ might enhance the hydrophilic property, which in turn might have improved the elasticity of the membrane, resulting in improved tensile strength (Supplementary Figure S5).

### Hemocompatibility assay

**Figure 5:**
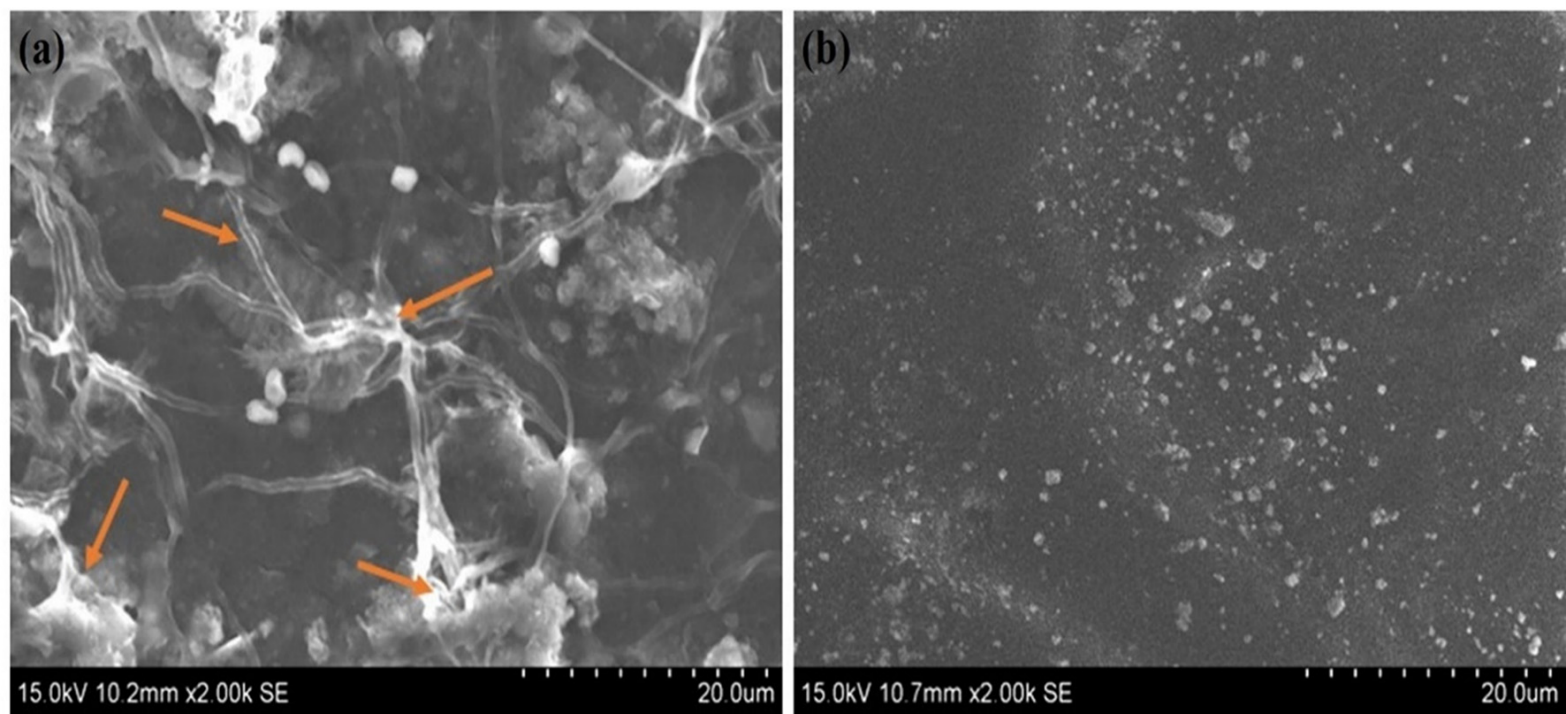
Scanning Electron Microscopy (SEM) of SPEEK and Ca-SPEEK in contact with blood In this figure are depicted the (**A**) SEM images of SPEEK and (**B**) Ca-SPEEK after treatment with blood. It shows fibrin meshes and protein adsorption (orange arrows) on both the polymer membranes wherein fibrin meshes are seen in the case of SPEEK, indicating the formation of clot, which is absent in Ca-SPEEK.

The first response for a biomaterial upon contact with blood is the adsorption of proteins onto its surface and contact with blood is the adsorption of proteins on to its surface, which is why it is essential to evaluate the hemocompatibility of the material used. Initially, these adsorbed proteins activate the platelets, which causes thrombus formation. The platelet adhesion and formation of a fibrin network in SPEEK were comparatively higher than in Ca-SPEEK when the SPEEK and Ca-SPEEK materials were tested for blood compatibility using SEM (Figure 5A,B). From Figure 5A,B, it can be inferred that the formation of 3D fibrin mesh is required for blood coagulation. Fibrin meshes, an essential factor for blood clots, increase platelet aggregation (as shown in Figure 5A) in the case of SPEEK membranes [22–24]. In contrast, fibrin meshes were absent in the case of Ca-SPEEK membranes, which could be attributed to the interaction of calcium and sulphonyl groups that mask the effect of both calcium and SPEEK in inducing the blood clot.

Similarly, based on the adsorbed weight of the blood clot, the compatibility of the material synthesized was studied, and the results of the study showed that the weight of the blood clot formed in the case of SPEEK was significantly higher when calcium was bound to the membrane (Table 1).

**Table 1:** Percentage of clot measured quantitatively after immersion of membranes in blood

Sample	Weight of blood clot (%)
**SPEEK**	21 ± 0.5 *
**Ca-SPEEK**	13 ± 0.5

Significantly higher than Ca-SPEEK (*P*<0.05, unpaired Student’s *t*-test).

The table shows the percentage of clot measured quantitatively after immersion of membranes in blood. The results of the study show that the weight of the blood clot formed in the case of SPEEK was significantly higher when calcium was bound to the membrane. The Ca-SPEEK exhibited 13 ± 0.5% clot with low fibrin mesh formation compared with 21 ± 0.5% in SPEEK (*P*<0.05, unpaired Student’s *t*-test) (see text and Figure 5).

### Protein adsorption

**Figure 6:**
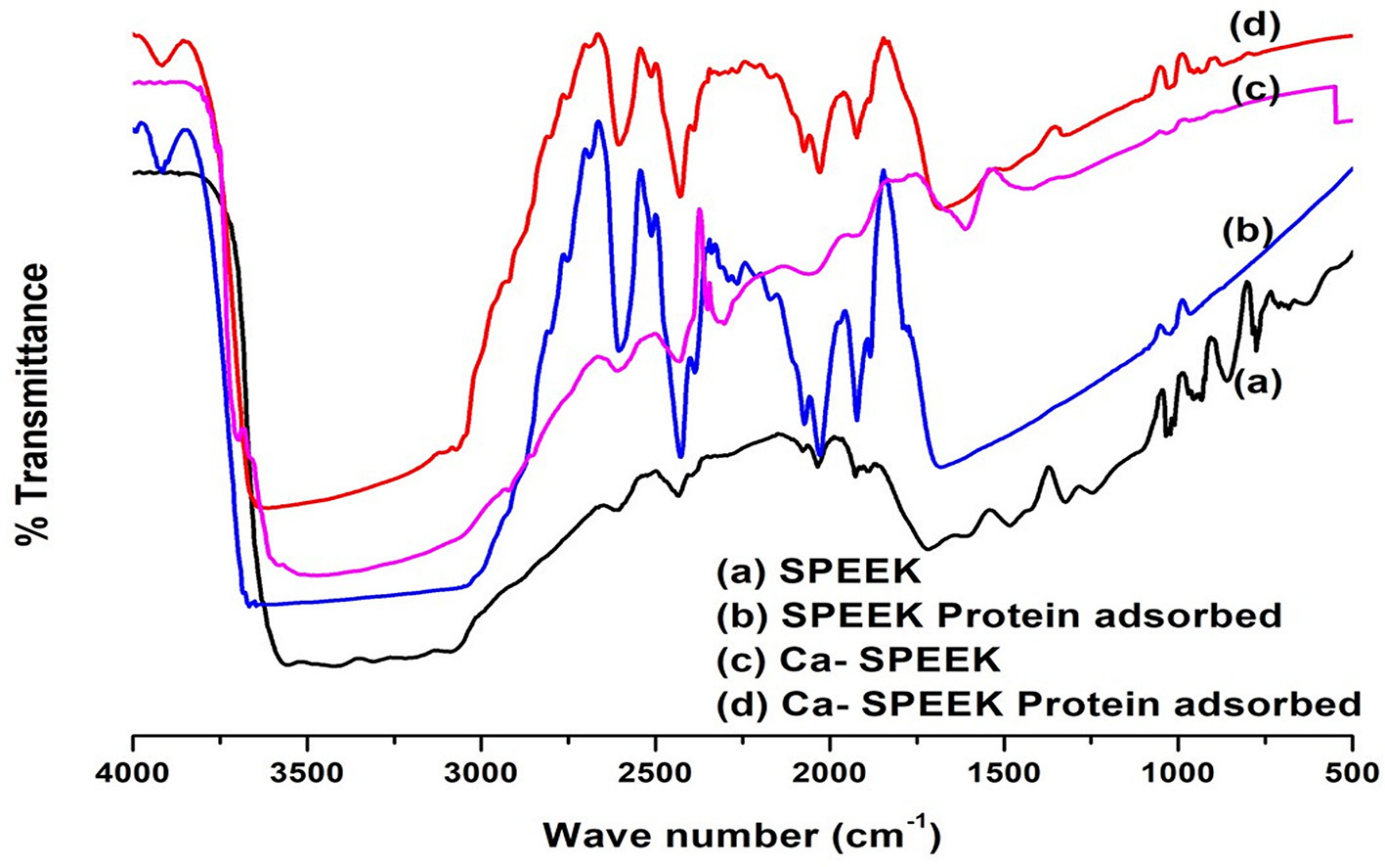
Fourier-transform infrared spectroscopy (FTIR) of SPEEK and Ca-SPEEK before and after protein adsorption FTIR spectrum of (**A,B**) SPEEK before and after protein adsorption. (**C,D**) Ca-SPEEK before and after protein adsorption. FTIR is a technique used to obtain an infrared spectrum of absorption or emission of a solid, liquid, or gas (see text).

The adsorption of protein on the surface of membranes was studied using FTIR spectra before and after protein adsorption. The FTIR spectra are shown in Figure 6A,B, which display the SPEEK membrane spectra, and Figure 6C,D shows the spectra of Ca-SPEEK before and after protein adsorption. The broadband observed in the high energy region at 3400 cm^−1^ was due to the OH group in sulfonic acid (SO_3_H), confirming the sulfonation of PEEK. The appearance of characteristic solid peaks at 1255, 1080, and 1020 cm^−1^ were assigned to the symmetric and asymmetric O=S=O stretching vibrations, confirming that the polymer PEEK was sulfonated. The peak at 1690 cm^−1^ indicates the presence of C–O stretching. The peak observed at 1255 cm^−1^ in Figure 6D indicated that the peak for O=S=O had shifted to a lower frequency due to Ca^2+^ and SO^3−^ interaction. The degree of sulfonation was in line with our earlier studies and confirmed through ^1^H NMR ^18^. The peaks seen in the region of 3914 cm^−1^ in Figure 6B,D are characteristic of N–H stretching.

The relatively more intense peak observed in Figure 6B than in (D) showed that Ca-SPEEK membranes adsorbed less protein than SPEEK membranes. The change in the peak was caused by the effect of Ca(OH)_2_ treatment on the polymer specimens’ protein adsorption characteristics. The decrease in protein adsorption in Ca-SPEEK can be attributed to an increase in membrane hydrophilicity after Ca(OH)_2_ treatment. It has been reported that hydrophobic materials adsorb more protein than hydrophilic polymer surfaces [20–27,29].

**Figure 7:**
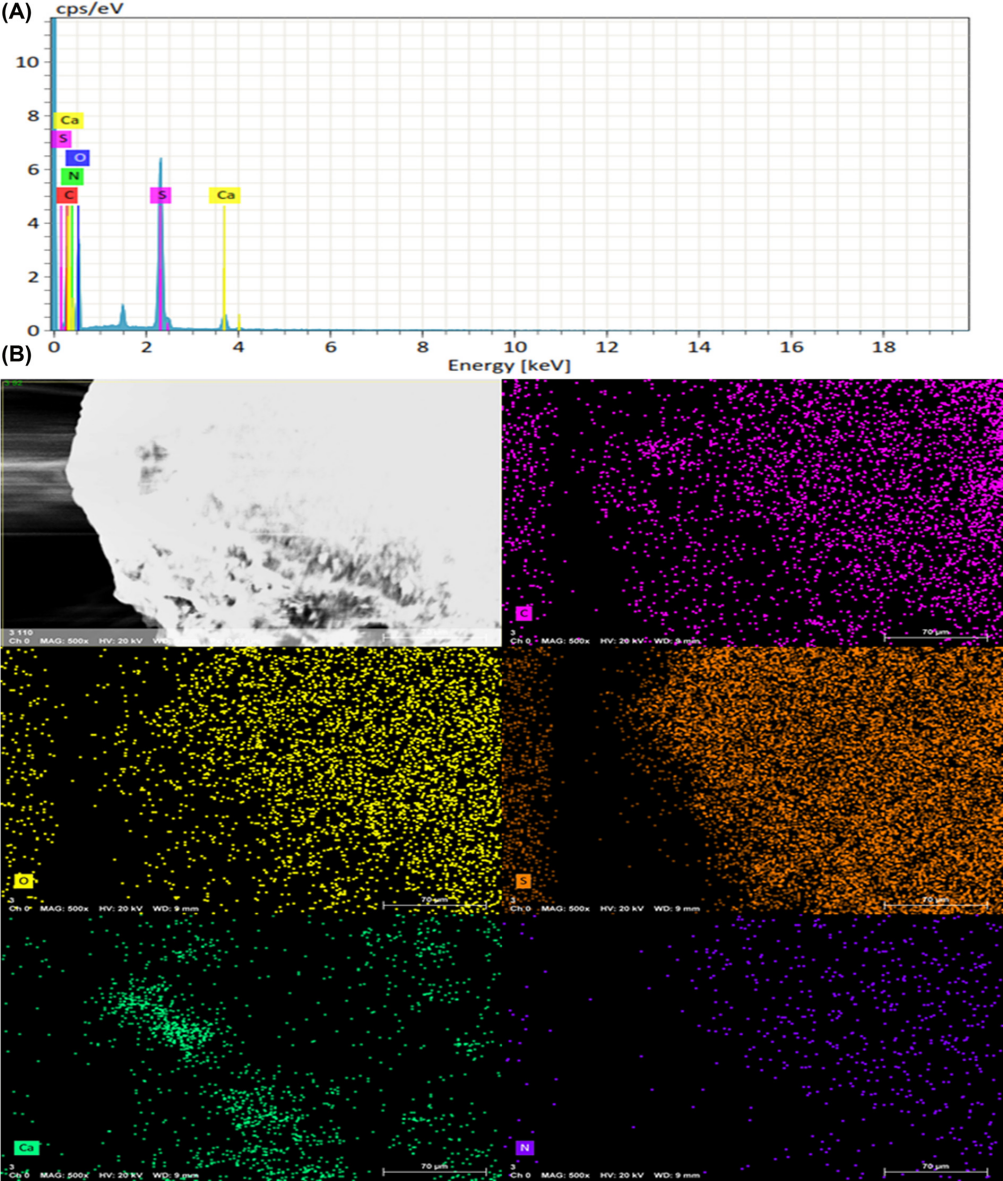
Energy-dispersive X-ray spectroscopy (EDS) of Ca-SPEEK in Trypsin This figure depicts the EDS (**A**) Spectra and (**B**) Mapping of Ca-SPEEK immersed in trypsin solution (see text).

In addition, Figure 7 shows the EDX spectra of Ca-SPEEK membrane samples immersed in trypsin solution. The spectra showed the weight percentage of nitrogen in both the treated and untreated samples was nearly equal (6.03% for Ca-SPEEK and 6.63% for SPEEK), indicating that SPEEK adsorbed slightly more protein. The calcium and phosphate groups were found to be in higher percentages in the case of Ca-SPEEK membranes than those in SPEEK.

### Inflammatory response

**Figure 8:**
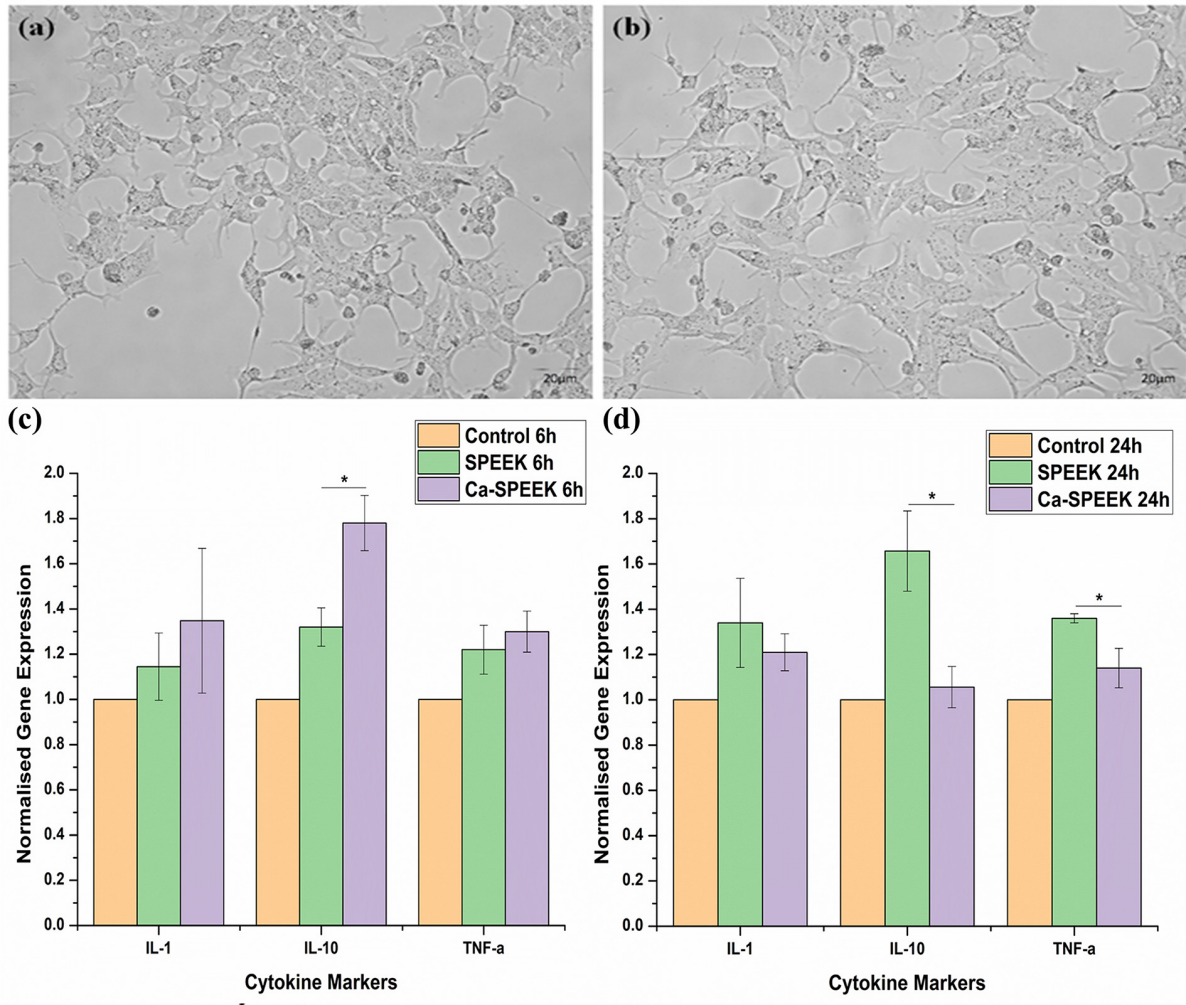
Cell morphology and cytokine profiles of BMDMS This figure shows the BMDMS cells morphology (**A**) before and (**B**) after treatment with Ca-SPEEK membrane and (**C,D**) gene expression of cytokine markers, including IL-1, IL-10, and TNF-α of SPEEK after 6 and 24 h, respectively (* denotes *P*<0.05, unpaired Student’s *t*-test). BMDMS refers to macrophage cells that are generated in a laboratory from mammalian bone marrow cells. BMDMs can differentiate into mature cells (macrophages) in the presence of growth factors and other signaling molecules (see text).

Generally, when a particle or any material interacts with cells, it is usually accompanied by a change in cellular morphology, the release of cytokines, and other immunogenic responses. Hence, cellular morphology and cytokine release become essential in evaluating the inflammatory response. However, the morphological impact of Ca-Speak on BMDMS before and after treatment showed no morphological change, suggesting that our composite membrane did not affect the morphology of cells when used (Figure 8A,B). Furthermore, the results were in pair with our inflammatory response study, which showed that the up-regulation of cytokine markers was lower in both SPEEK and Ca-SPEEK compared with that of the control group.

## Discussion

The increase in density of apatite formation in Ca-SPEEK membranes compared with SPEEK was due to calcium on the surface of the membrane. Indeed, it induces faster nucleation and growth of apatite crystals over a larger surface area. The bioactivity observed with the SPEEK-based membranes in this study was similar to that observed in previous studies [11]. While Aravind et al. (2010) reported that SPEEK membranes only showed bioactivity when hydroxyapatite was added, incorporating Ca-induced dense apatite formation is critical [11]. The water uptake results complemented the bioactivity results with increased apatite formation. An important finding was that the weight of the blood clot was low for Ca-SPEEK, although reports show calcium as a powerful clotting agent. The lower levels of the blood clots were attributed to the formation of the calcium-sulfonic (SO_3_H) bond, which could be a possible reason for the reduction in blood clots due to calcium. During the coagulation of blood, calcium, being a coagulation factor, helps bind these proteins and factors to the phospholipids, thereby aiding blood coagulation. It is also possible that the sulfonate salt formed between the SPEEK and Ca is responsible for the anti-coagulation effect, as some sulfonate salts are widely used anti-coagulants [25,26]. Calcium is known to enhance fibrin aggregation by binding covalently with fibrin polymers [27]. However, in the present study, calcium is not available as a free element to bind with the fibrin polymers. Hence, the effect of calcium in promoting coagulation is negated due to the binding of calcium with the sulfonic groups of SPEEK. Moreover, Ca-SPEEK displayed an increase in the hydrophilicity of the membranes, as given in Table S1 [28], which could contribute to the observed anti-coagulant effect. Despite having some evidence of sulfonate salts being used as anti-coagulants, the exact mechanism behind the anti-coagulation in our study is unclear. Nevertheless, this finding is significant as it implies that calcium binding to SPEEK membranes shows a considerable improvement in hemocompatibility compared to normal SPEEK membranes.

As expected, pro- and anti-inflammatory cytokines, IL-1, TNF-α, and IL-10, were moderately up-regulated in BMDMs by SPEEK and Ca-SPEEK compared with untreated cells (Figure 8C,D), as is typical when foreign material is introduced into the cellular environment. Incubation of BMDMs for 6 h with the membranes induced a slight up-regulation of IL-1 and TNF-α, but there was no difference between the SPEEK and Ca-SPEEK treatments. In contrast, there was a significantly increased expression of the anti-inflammatory IL-10 in the case of Ca-SPEEK compared with the SPEEK group (Figure 8C). Following 24-h incubation, the IL-10 expression significantly decreased compared with the 6-h level in the case of the Ca-SPEEK-treated cells, while its expression increased significantly in the SPEEK-treated BMDMS compared with the 6-h samples. TNF-α expression was also markedly lower in BMDMs treated with Ca-SPEEK for 24 h compared with SPEEK-treated cells (Figure 8D). These results indicate Ca-SPEEK induces an early anti-inflammatory response in the BMDMs, which might contribute to the lower TNF-α production observed in the 24-h Ca-SPEEK-treated cells as compared with the 24-h SPEEK group. The study results reported by researchers involved SPEEK-based membranes tested on rabbit models for inflammation. This could be considered supporting evidence for SPEEK compatibility, though it is not the focus of our current study [30]. In the case of Ca-SPEEK, the up-regulation of IL-10 after 6 h was almost double when compared with the control group, which was reduced after 24 h (Figure 8D). The early regulation of IL-10 in the case of Ca-SPEEK may be attributed to the downregulation of the inflammatory markers IL-1 and TNF-α at 24 h. In addition, the upregulation of IL-10 shows that Ca-SPEEK has a faster inflammatory program than SPEEK. However, several genes may be involved in regulating the increased expression of IL-10, which is beyond the scope of our current study [31].

In addition, the ROS assay results (Supplementary Figure S3) of the Ca-SPEEK showed that the material does not induce any stress to the cells, thereby being in line with the inflammatory studies. It is also a worthy observation that the other two cytokine expressions were comparatively similar to that of SPEEK and slightly higher than the control, which may be negligible since the up-regulation was reduced after 24 h of incubation, as shown in Figure 8D. The regulation of these pro-inflammatory cytokines was lower in Ca-SPEEK compared to SPEEK due to the incorporation of Ca into the SPEEK membrane. This hypothesis of reduced inflammation after 24 h in Ca-incorporated SPEEK is similar to the one observed by DeSousa et al. in 2016, wherein, again, the study analysis is beyond the scope of this study. The study does show, however, that the expression of ICAM-1 by endothelial cells decreases with calcium incorporation and thus acts as an anti-inflammatory response, which may be the reason for a lower level of cytokine expression in Ca-SPEEK compared to SPEEK after 24 h in BMDMS macrophages (Figure 8D) [32–34]. Wei et al. (2019) have reported that the SPEEK, due to its porous surface, lowered the TNF-α concentrations to dramatically lower levels and enhanced the concentration of IL-10. In addition, their study revealed enhanced differentiation of M2 macrophages. On the other hand, the un-sulphonated PEEK significantly increased the inflammatory response mediated by M1 macrophages. Moreover, *in vivo*, their studies proved that the sulphonation of PEEK will have a significant anti-inflammatory role, which is in line with our studies. Another study by Buck et al. (2021) showed that the COOH modification of PEEK has significantly reduced inflammation after 3 days of implantation by down-regulating most of the proinflammatory markers [35–36]. It may hence be noted that, our results on anti-inflammatory and hemocompatibility studies of surface modified SPEEK and Ca-SPEEK were comparable and similar to those observed by the other researchers. However, in addition to the above findings, the authors opine that further research including MTT assay on osteoblasts and mouse fibroblasts as well as activated macrophages may be warranted to further substantiate the above discussed findings on SPEEK and Ca-SPEEK membranes. Consequently, in the present scenario, Ca incorporation into SPEEK membranes proved to be an alternative to SPEEK to enhance the bioactivity, reduce inflammation, and make it a compatible material when applied *in vivo*.

## Conclusion

In the present study, we successfully synthesized and fabricated SPEEK, which was modified by incorporating Ca(OH)_2_ solution to form Ca-SPEEK. The *in vitro* bioactivity studies revealed that Ca in the Ca-SPEEK membranes positively influences the nucleation size and the number of hydroxyapatite crystals. Furthermore, the prepared membranes showed higher protein adsorption and lesser fibrin mesh formation than SPEEK membranes, exhibiting their effectiveness as a blood-compatible biomaterial. Incorporating calcium into the membrane modifies its surface property by increasing its hydrophilicity. It may enhance their bioactivity and lead to higher protein adsorption. The altered surface energy may also be responsible for the difference in protein adsorption and hemocompatibility between SPEEK and Ca-SPEEK [25]. This implies that we get enhanced bioactivity upon increasing the calcium content in the membranes, which makes it favorable for application as a blood-contacting material. Similarly, the inflammatory response of Ca-SPEEK in bone marrow-derived macrophage cells from mice showed a lower number of inflammatory markers than SPEEK. Therefore, Ca-SPEEK membranes appear promising for application in orthopedics because of their enhanced bioactivity, hemocompatibility, and inflammatory response. In addition, it might open doors for more applications *in vivo*.

## Supplementary Material

Supplementary Figures S1-S6 and Table S1

## Data Availability

The data supporting this study’s findings are available from the corresponding author upon reasonable request.

## Abbreviations

DMEM: Dulbecco’s Modified Eagle Medium
EDS: Energy-dispersive X-ray spectroscopy
NMP: N-Methyl Pyrrolidone
PEEK: Polyether ether ketone
SBF: simulated body fluid
SEM: Scanning Electron Microscopy
SPEEK: Sulfonated polyether ether ketone
XRD: X-ray diffraction

## References

[1] Yutitum K. and Khantachawana A. (2020) Forming and coating of hydroxyapatite on PEEK substrate for orthopedic implants. Int. J. Appl. Physical Sci. 6, 16–22 10.20469/ijaps.6.50003

[2] Shanmuga Sundar S. and Sangeetha D. (2012) Fabrication and evaluation of electrospun collagen/poly (N-isopropyl acrylamide)/chitosan mat as blood-contacting biomaterials for drug delivery. J. Mater. Sci.: Mater 23, 1421–1430 10.1007/s10856-012-4610-x 22476650

[3] Saravanabhavan S.S. and Dharmalingam S. (2013) Fabrication of polysulphone/hydroxyapatite nanofiber composite implant and evaluation of their in vitro bioactivity and biocompatibility towards the post-surgical therapy of gastric cancer. Chem. Eng. J. 234, 380–388 10.1016/j.cej.2013.08.076

[4] Bhat K.A., Prabhu N.V. and Sangeetha D. (2012) Polymer/silica composites fabricated by sol-gel technique for medical applications. Trends Biomater Artif Organs 26, 121–129 https://www.researchgate.net/profile/Prabhu-N-V/publication/228096193_PolymerSilica_Composites_Fabricated_by_Sol-Gel_Technique_for_Medical_Applications/links/09e4150eb14441c9d3000000/Polymer-Silica-Composites-Fabricated-by-Sol-Gel-Technique-for-Medical-Applications.pdf22754200

[5] Peng S., Feng P., Wu P., Huang W., Yang Y., Guo W. et al. (2017) Graphene oxide as an interface phase between polyetheretherketone and hydroxyapatite for tissue engineering scaffolds. Sci. Rep. 7, 1–14 10.1038/srep46604 28425470 PMC5397874

[6] Wang M.-C., Chen H.-T., Shih W.-J., Chang H.-F., Hon M.-H. and Hung I.-M. (2015) Crystalline size, microstructure and biocompatibility of hydroxyapatite nanopowders by hydrolysis of calcium hydrogen phosphate dehydrate (DCPD). Ceram. Int. 41, 2999–3008 10.1016/j.ceramint.2014.10.135

[7] Feng P., Niu M., Gao C., Peng S. and Shuai C. (2014) A novel two-step sintering for nano-hydroxyapatite scaffolds for bone tissue engineering. Sci. Rep. 4, 1–10 10.1038/srep05599 PMC4083286 24998362

[8] Elschner C., Noack C., Preißler C., Krause A., Scheler U. and Hempel U. (2015) In vitro response of human mesenchymal stromal cells to titanium coated peek films and their suitability for magnetic resonance imaging. J. Mater. Sci. Technol. 31, 427–436 10.1016/j.jmst.2014.10.012

[9] Tsukeoka T., Suzuki M., Ohtsuki C., Sugino A., Tsuneizumi Y., Miyagi J. et al. (2006) Mechanical and histological evaluation of a PMMA-based bone cement modified with γ-methacryloxypropyltrimethoxysilane and calcium acetate. Biomaterials 27, 3897–3903 10.1016/j.biomaterials.2006.03.002 16563499

[10] Aravind K. and Sangeetha D. (2015) Characterization and in vitro studies of sulfonated polyether ether ketone/polyether sulfone/nano hydroxyapatite composite. Int. J. Polym. Mater. Polym. Biomater. 64, 220–227 10.1080/00914037.2014.936594

[11] Shanmuga Sundar S. and Sangeetha D. (2012) Investigation on sulphonated PEEK beads for drug delivery, bioactivity and tissue engineering applications. J. Mater. Sci. 47, 2736–2742 10.1007/s10853-011-6100-9

[12] Leonor I., Kim H.-M., Balas F., Kawashita M., Reis R., Kokubo T. et al. (2007) Functionalization of different polymers with sulfonic groups as a way to coat them with a biomimetic apatite layer. J. Mater. Sci. Mater. Med. 18, 1923–1930 10.1007/s10856-007-3106-6 17554598

[13] Kamaraj J., Swaminathan E. and Dharmalingam S. (2009) Development and characterization of polymer ceramic composites for orthopedic applications. Trends Biomater. Artif Organs 22, 165–175, https://go.gale.com/ps/i.do?id=GALE%7CA192639997&sid=googleScholar&v=2.1&it=r&linkaccess=abs&issn=09711198&p=HRCA&sw=w&userGroupName=tel_oweb&isGeoAuthType=true&aty=geo

[14] Tande A.J. and Patel R. (2014) Prosthetic joint infection. Clin. Microbiol. Rev. 27, 302–345 10.1128/CMR.00111-13 24696437 PMC3993098

[15] Ikada Y. (1994) Surface modification of polymers for medical applications. Biomaterials 15, 725–736 10.1016/0142-9612(94)90025-6 7986935

[16] Roach P., Farrar D. and Perry C.C. (2005) Interpretation of protein adsorption: surface-induced conformational changes. J. Am. Chem. Soc. 127, 8168–8173 10.1021/ja042898o 15926845

[17] Siriphannon P., Kameshima Y., Yasumori A., Okada K. and Hayashi S. (2000) Influence of preparation conditions on the microstructure and bioactivity of α‐CaSiO3 ceramics: Formation of hydroxyapatite in simulated body fluid. J. Biomed. Mater. 52, 30–39 10.1002/1097-4636(200010)52:1<30::AID-JBM5>3.0.CO;2-Z 10906672

[18] Venkatesan P.N. and Dharmalingam S. (2017) Characterization and performance study of phase inversed Sulfonated Poly Ether Ether Ketone-Silico tungstic composite membrane as an electrolyte for microbial fuel cell applications. Renew. Energy 102, 77–86

[19] Oyane A., Kim H.M., Furuya T., Kokubo T., Miyazaki T. and Nakamura T. (2003) Preparation and assessment of revised simulated body fluids. J. Biomed. Mater. Res. A 65, 188–195 10.1002/jbm.a.10482 12734811

[20] Koutsopoulos S., Patzsch K., Bosker W.T. and Norde W. (2007) Adsorption of trypsin on hydrophilic and hydrophobic surfaces. Langmuir 23, 2000–2006 10.1021/la062238s 17279687

[21] Saravanabhavan S.S., Rethinasabapathy M., Zsolt S., Kalambettu A.B., Elumalai S., Janakiraman M. et al. (2019) Graphene oxide functionalized with chitosan based nanoparticles as a carrier of siRNA in regulating Bcl-2 expression on Saos-2 & MG-63 cancer cells and its inflammatory response on bone marrow derived cells from mice. Mater. Sci. Eng. 99, 1459–1468 10.1016/j.msec.2019.02.047 30889680

[22] Zhang L., Casey B., Galanakis D.K., Marmorat C., Skoog S., Vorvolakos K. et al. (2017) The influence of surface chemistry on adsorbed fibrinogen conformation, orientation, fiber formation and platelet adhesion. Acta Biomater. 54, 164–174 10.1016/j.actbio.2017.03.002 28263863

[23] Weber M., Steinle H., Golombek S., Hann L., Schlensak C., Wendel H.P. et al. (2018) Blood-contacting biomaterials: in vitro evaluation of the hemocompatibility. Front. Bioeng. Biotechnol. 6, 99 10.3389/fbioe.2018.00099 30062094 PMC6054932

[24] Aguilar M.R., Rodríguez G., Fernández M., Gallardo A. and Román J.S. (2002) Polymeric active coatings with functionality in vascular applications. J. Mater. Sci. Mater. Med. 13, 1099–1104 10.1023/A:1021100916920 15348650

[25] Kindness G., Williamson F.B. and Long W.F. (1979) Effect of polyanetholesulphonic acid and xylan sulphate on antithrombin III activity. Biochem. Biophys. Res. Commun. 88, 1062–1068 10.1016/0006-291X(79)91516-X 223567

[26] Palarasah Y., Skjoedt M.-O., Vitved L., Andersen T.E., Skjoedt K. and Koch C. (2010) Sodium polyanethole sulfonate as an inhibitor of activation of complement function in blood culture systems. J. Clin. Microbiol. 48, 908–914 10.1128/JCM.01985-09 20042630 PMC2832435

[27] Ratner B.D. and Latour R.A. (2020) Role of water in biomaterials. Biomaterials Science, pp. 77–82, Elsevier 10.1016/B978-0-12-816137-1.00007-6

[28] Butera D. and Hogg P.J. (2020) Fibrinogen function achieved through multiple covalent states. Nat. Commun. 11, 1–10 10.1038/s41467-020-19295-7 33122656 PMC7596563

[29] Chittur K.K. (1998) FTIR/ATR for protein adsorption to biomaterial surfaces. Biomaterials 19, 357–369 10.1016/S0142-9612(97)00223-8 9677150

[30] Aravind K., Sundar S.S. and Sangeetha D. (2014) In vivo studies of sulphonated polyether ether ketone based composite bone graft materials. Trends Biomater. Artif Organs 28, 52–58, https://go.gale.com/ps/i.do?id=GALE%7CA430547965&sid=googleScholar&v=2.1&it=r&linkaccess=abs&issn=09711198&p=AONE&sw=w&userGroupName=tel_oweb&aty=ip 10.1016/S0142-9612(97)00223-8

[31] Lin H., Joehanes R., Pilling L.C., Dupuis J., Lunetta K.L., Ying S.-X. et al. (2014) Whole blood gene expression and interleukin-6 levels. Genomics 104, 490–495 10.1016/j.ygeno.2014.10.003 25311648 PMC4262595

[32] DeSousa J., Tong M., Wei J., Chamley L., Stone P. and Chen Q. (2016) The anti-inflammatory effect of calcium for preventing endothelial cell activation in preeclampsia. J. Hum. Hypertens. 30, 303–308 10.1038/jhh.2015.73 26155993

[33] Chen Q., Tong M., Wu M., Stone P.R., Snowise S. and Chamley L.W. (2013) Calcium supplementation prevents endothelial cell activation: possible relevance to preeclampsia. J. Hypertens. 31, 1828–1836 10.1097/HJH.0b013e328362ba1a 23822977

[34] Bastie C.C., Gaffney-Stomberg E., Lee T.-W.A., Dhima E., Pessin J.E. and Augenlicht L.H. (2012) Dietary cholecalciferol and calcium levels in a Western-style defined rodent diet alter energy metabolism and inflammatory responses in mice. J. Nutr. 142, 859–865 10.3945/jn.111.149914 22437564 PMC3327744

[35] Wei R., Wu J. and Li Y. (2019) Macrophage polarization following three-dimensional porous PEEK. Mater. Sci. Eng. C 104, 109948 10.1016/j.msec.2019.109948 31499957

[36] Buck E., Lee S., Stone L.S. and Cerruti M. (2021) Protein adsorption on surfaces functionalized with COOH groups promotes anti-inflammatory macrophage responses. ACS Appl. Mater. Interfaces 13, 7021–7036 10.1021/acsami.0c16509 33539069

