# Supplementary material for: Bioactivity, hemocompatibility, and inflammatory response of calcium incorporated sulfonated polyether ether ketone on mouse-derived bone marrow cells
Source: Biosci Rep. 2024 Jun 26;44(6):BSR20232162. doi: 10.1042/BSR20232162 (PMC11208130; doi:10.1042/BSR20232162)
Supplement: Supplementary Figures S1-S6 and Table S1 [file BSR-2023-2162_supp.pdf]

7. Supplementary Information

7.1 Materials and Methods

The reaction mechanism of conversion of Poly ether ether ketone to Sulphonated poly ether ether ketone is shown in figure S1. The membranes synthesized before and after incorporation of Ca onto SPEEK was observed under optical microscope and found to be as shown in figure S2.

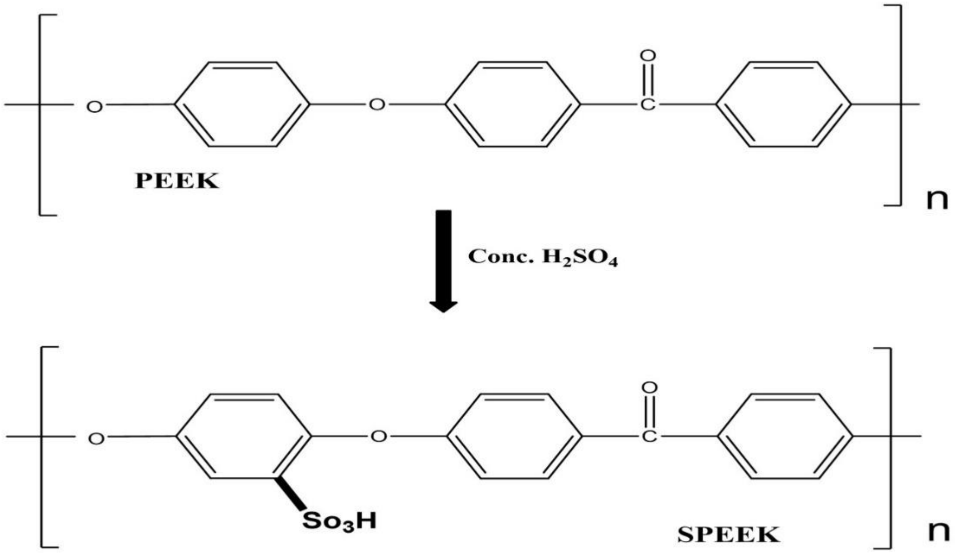

Figure S 1. Chemical Sulfonation of PEEK

This illustration shows the sulfonation of PEEK.

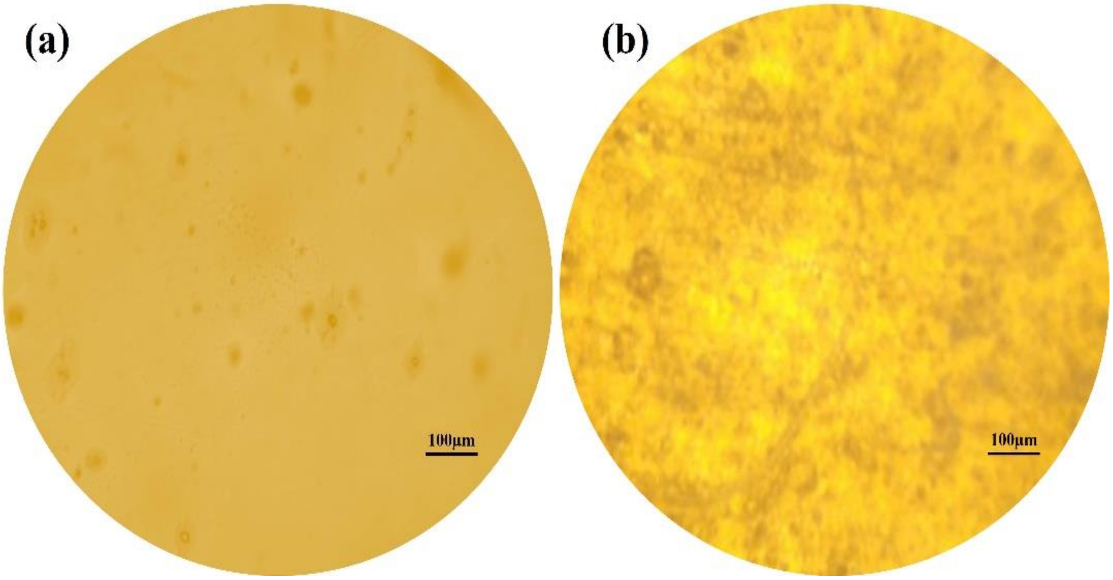

Figure S 2. Optical Polarising Microscope Images.

(a) OPM image of SPEEK and (b) Ca-SPEEK

7.2. EDS of SPEEK before Immersion in SBF solution

EDS spectra of SPEEK confirmed the presence of sulphonation peeks in the spectra and in the mapping. The presence of carbon, oxygen and sulphur groups were confirmed from the EDS spectra and mapping (Figure S3).

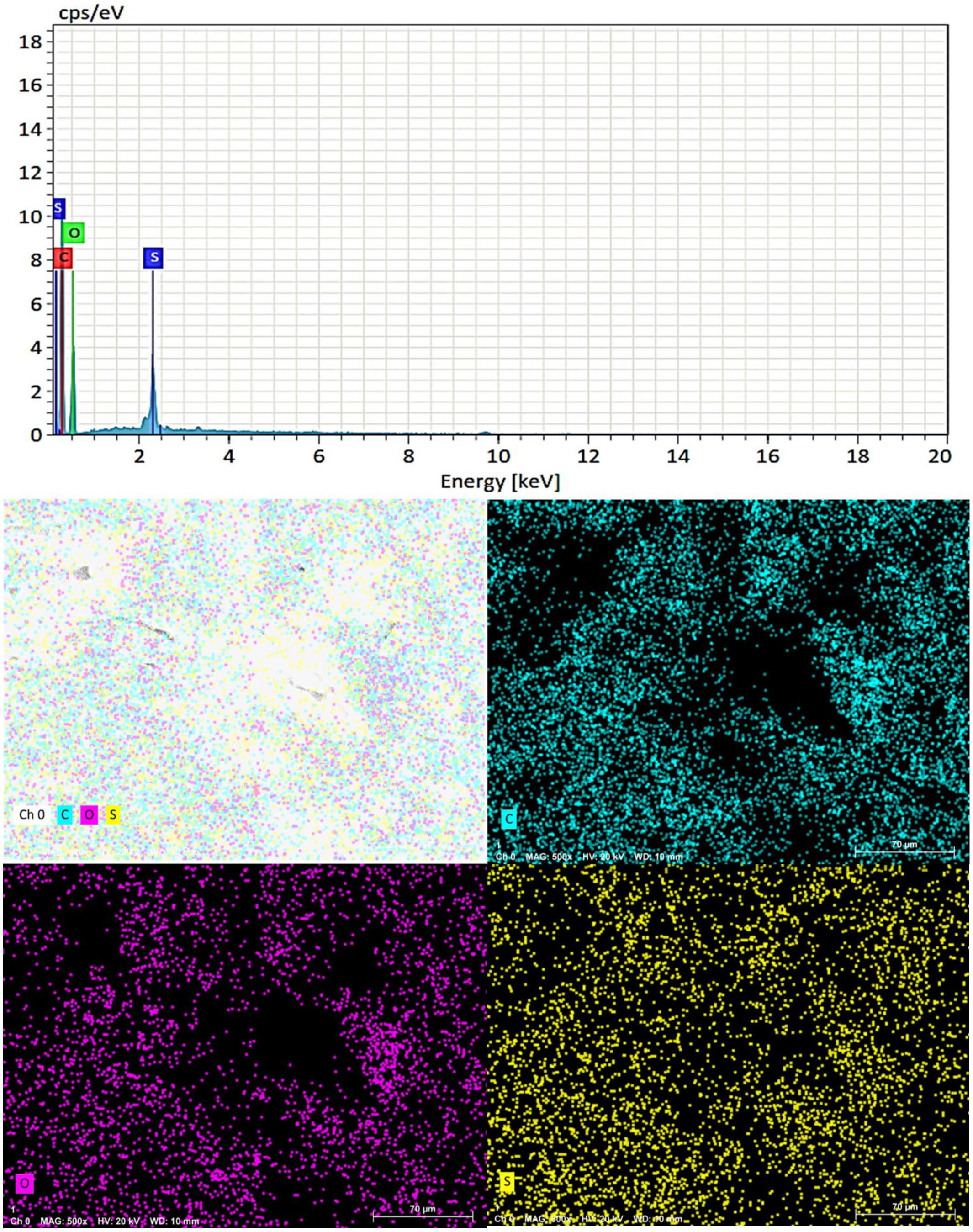

Figure S3. EDS Spectra and Mapping.

This figure depicts EDS spectra and mapping of a prepared SPEEK membrane.

7.3. EDS of Ca-SPEEK before Immersion in SBF solution.

EDS spectra of Ca-SPEEK confirmed the presence of sulphonation peeks in the spectra and in the mapping. The presence of carbon, oxygen and sulphur groups were confirmed from the EDS spectra and mapping (Figure S4).

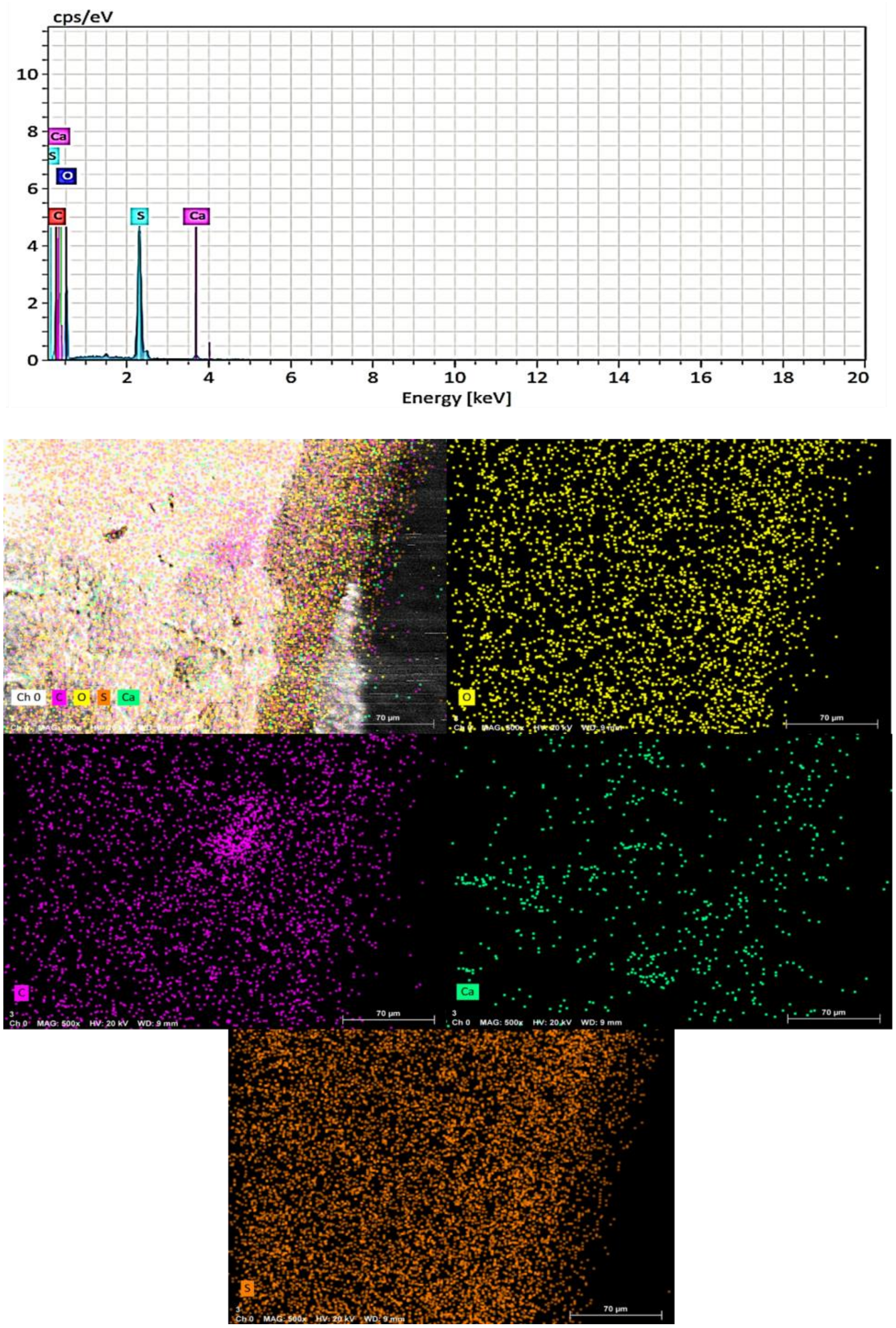

Figure S4. EDS Spectra and Mapping.

EDS spectra and mapping of as prepared Ca-SPEEK membrane

7.4 Water uptake Studies

The values of water uptake of the SPEEK and Ca-SPEEK membranes are given in Table 1. The presence of sulfonic acid groups in SPEEK makes it hydrophilic when compared to that of PEEK. Similarly, the incorporation of calcium increased the water absorption property of the Ca-SPEEK composite membrane. The increase in water uptake is crucial since this increased the apatite nucleation on the membrane surface when immersed in SBF as discussed below.

Table S1. Water Uptake.

This table shows the water uptake characteristic of both SPEEK and Ca-SPEEK

Membrane	Water uptake (%)	Thickness
**SPEEK**	15.85	0.018 (±0.002)
**Ca-SPEEK**	17.45	0.019 (±0.001)

7.5 Tensile Strength

The tensile strength of SPEEK was enhanced with the incorporation of Ca which can be observed from figure S5.

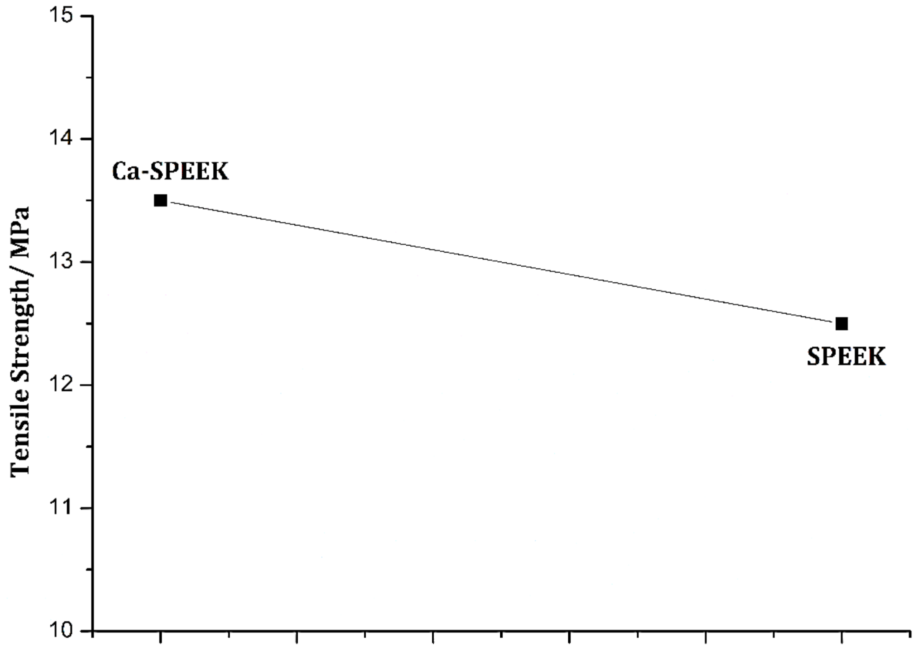

Figure S5. Tensile Strength of SPEEK and Ca-SPEEK membrane.

The figure depicts the Tensile Strength of SPEEK and Ca-SPEEK membrane.

7.6 ROS Assay

The result of ROS assay showed that the level of oxygen species was not enhanced after exposure to Ca-SPEEK which was in par with our previous studies.

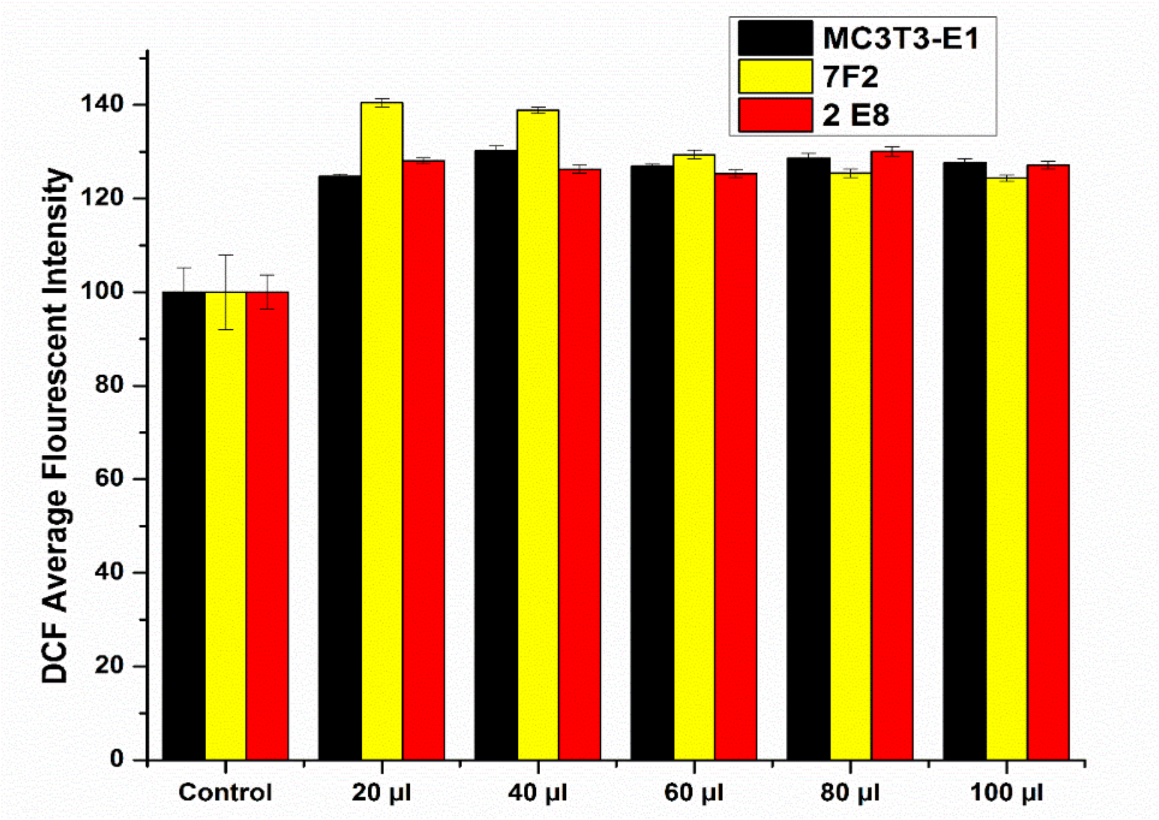

Figure S6. Reactive Oxygen Species Assay Results.

This figure shows the oxidative stress due to exposure of Ca-SPEEK material.
